# Assessing a partnership-based model of surgical education in the Global South: a mixed methods study of the University of Global Health Equity, Rwanda

**DOI:** 10.1186/s12893-025-02996-5

**Published:** 2025-07-03

**Authors:** Naol Belema Gemechu, Gatwiri Murithi, Derbew Fikadu Berhe, Betel Amdeslassie Fenta, Amanuel Adane Bitew, Tairu Fofanah, Barnabas Tobi Alayande, Abebe Bekele, Geoffrey Anderson, Robert Riviello

**Affiliations:** 1 Center for Equity in Global Surgery, University of Global Health Equity, Kigali, Rwanda; 2 ALERT Comprehensive Specialized Hospital, Addis Ababa, Ethiopia; 3 Center for Public Health and Development, Nairobi, Kenya; 4 Program in Global Surgery and Social Change, Harvard Medical School, Boston, MA USA; 5 St. Peter Specialized Hospital, Addis Ababa, Ethiopia; 6 Ministry of Health Sierra Leone, Free Town, Sierra Leone

**Keywords:** Academic partnerships, Surgical education, Partnership-based medical education, Global surgery, Equity, Medical, Education

## Abstract

**Background:**

Workforce shortages, resource limitations, and inadequate capacity in African higher education institutions are significant challenges that hinder their global competitiveness in generating knowledge products. Academic partnerships have been established to address these gaps. It is essential to evaluate these partnerships to ensure they align with principles of ethics, equity, reciprocity, and the achievement of shared goals. The University of Global Health Equity (UGHE) is an institution that employs a partnership-based model to deliver high-quality surgical education. The aim of the study was to assess its partnership based surgical education programs.

**Methods:**

This study was conducted at UGHE using a sequential exploratory mixed-methods design that incorporates perspectives of learners, facility and partners. Qualitative interviews were conducted with students, faculty, and partners involved in UGHE’s surgical education programs within the last three years. Thematic analysis was employed to interpret the interview data. Quantitative data were summarized using descriptive statistics and presented in charts and tables with integration in a joint display.

**Results:**

Twenty-one interviews were conducted, revealing 4 key themes from the thematic analysis: (1) A needs-based approach is used to determine the suitability of partnerships (2), UGHE and its partners worked towards equitable outcomes (3), Positive outcomes of the partnership model (4) Challenges faced in delivering surgical education using a partnership model and proposed solutions. Most participants viewed the model positively, identifying benefits such as diverse exposure, improved student experiences, faculty development, and technology transfer. However, institutional and systemic gaps that limit maximum benefits were noted. The quantitative survey had a 42% response rate with 31 responses from undergraduate and postgraduate students. All students agreed that didactic and simulation sessions led by UGHE partners enhanced their learning. A significant difference (*p* <.001) was found between postgraduate and undergraduate students’ responses regarding the adequacy of time for partner-facilitated sessions.

**Conclusions:**

The results underscore the significant positive impact of UGHE’s institutional partnership-based model in delivering surgical education, especially in enhancing student learning and faculty capacity. However, communication gaps, lack of resources, and time prevent the partnership-based model from reaching full potential.

**Supplementary Information:**

The online version contains supplementary material available at 10.1186/s12893-025-02996-5.

## Introduction

Partnerships in medical education are relationships resembling a legal association that usually involves close cooperation between parties with specified and joint rights and responsibilities in providing medical training and education [1]. This term is often used interchangeably with collaboration, especially in research and education contexts. The foundation of successful partnerships rests on good personal relationships with key stakeholders, trust, and effective communication [2]. Partners typically come together with a shared objective, driven by the desire to achieve common and specified goals [3]. As collaborations evolve, they may lead to more formal agreements that strengthen long-term engagement [4].

Global health education partnerships help provide practical experiences that enhance classroom-based learning, ultimately improving healthcare delivery in low- and middle-income countries (LMICs) [5]. Partnerships can take many forms, including leadership secondment, student, and faculty training, and infrastructure development [6]. These partnerships are especially valuable for building local capacity in LMICs, where institutions may lack the resources to independently provide high-quality medical education [7].

Global health partnerships for medical education aim to address emerging and existing disease burdens by training a competent workforce. In Africa, higher education institutions form partnerships to tackle institutional challenges, enhance research, and improve teaching quality [8]. Partnerships to enhance the delivery of surgical education are not a new phenomenon. Collaborations between institutions, such as the neurosurgery residency partnership in Ethiopia and the surgical and anesthesia training partnership at Makerere University in Uganda have successfully bridged gaps in faculty development and resource sharing [9]. Such global partnerships often lead to significant benefits such as knowledge sharing, clinical practice improvement, and infrastructure development [10]. However, partnerships in global health are not without challenges. Early academic partnerships, especially those between Global North and Global South institutions, often suffered from power imbalances and unidirectional knowledge transfer, with the High-Income Country institutions often benefiting disproportionately [11]. The lack of shared ownership, mutual respect, and equitable distribution of resources has led to criticisms of many partnerships as surgical education partnerships are not always mutually beneficial [12].

The University of Global Health Equity (UGHE) is an institution in Rwanda working to improve surgical education in the region. The university utilizes a partnership-based model to facilitate the delivery of quality medical education for its students. UGHE has partnered with both Global North and Global South institutions to enhance its curriculum delivery and to give up-to-date medical education that is supported by technology [13]. The partners help in surgical clerkship program provision for medical undergraduate students and to facilitate the masters in global surgery program. Moreover, the university has recently started the delivery of a global surgery advocacy program through its partnerships.

Given the complexity, variability, and dynamic nature of such global health partnerships, it is crucial to assess them regularly to ensure they align with ethical principles, equity, and shared goals. While there is no universally accepted framework, principles like trust, transparency, and shared ownership guide partnership assessments [14, 15] Current literature shows that evaluating the impact of partnerships at the individual, programmatic, and institutional levels helps ensure that collaborations are effective, beneficial, and sustainable [10].

In the context of academic partnerships, researchers have attempted to assess learner perspectives on the utility of shared resources is vital for ensuring that partnerships contribute to the improvement of medical education quality [16]. This paper aims to add to literature on programmatic evaluation of partnership-based surgical education by providing a holistic assessment of UGHE’s academic partnerships.

Specific aims of this mixed methods study are:

1. To provide a comprehensive understanding of the effectiveness of UGHE’s partnership-based models in achieving educational goals.
2. To identify successful partnership strategies, ethical challenges, and best practices in UGHE’s collaborative surgical education. This study will evaluate the mutuality and adherence to principles of ethics and equity in partnerships.

We will provide recommendations for optimizing existing partnerships and guidance for establishing new collaborations by incorporating the perspectives of different stakeholders such as learners, faculty, and partners.

## Methods

**Fig. 1:**
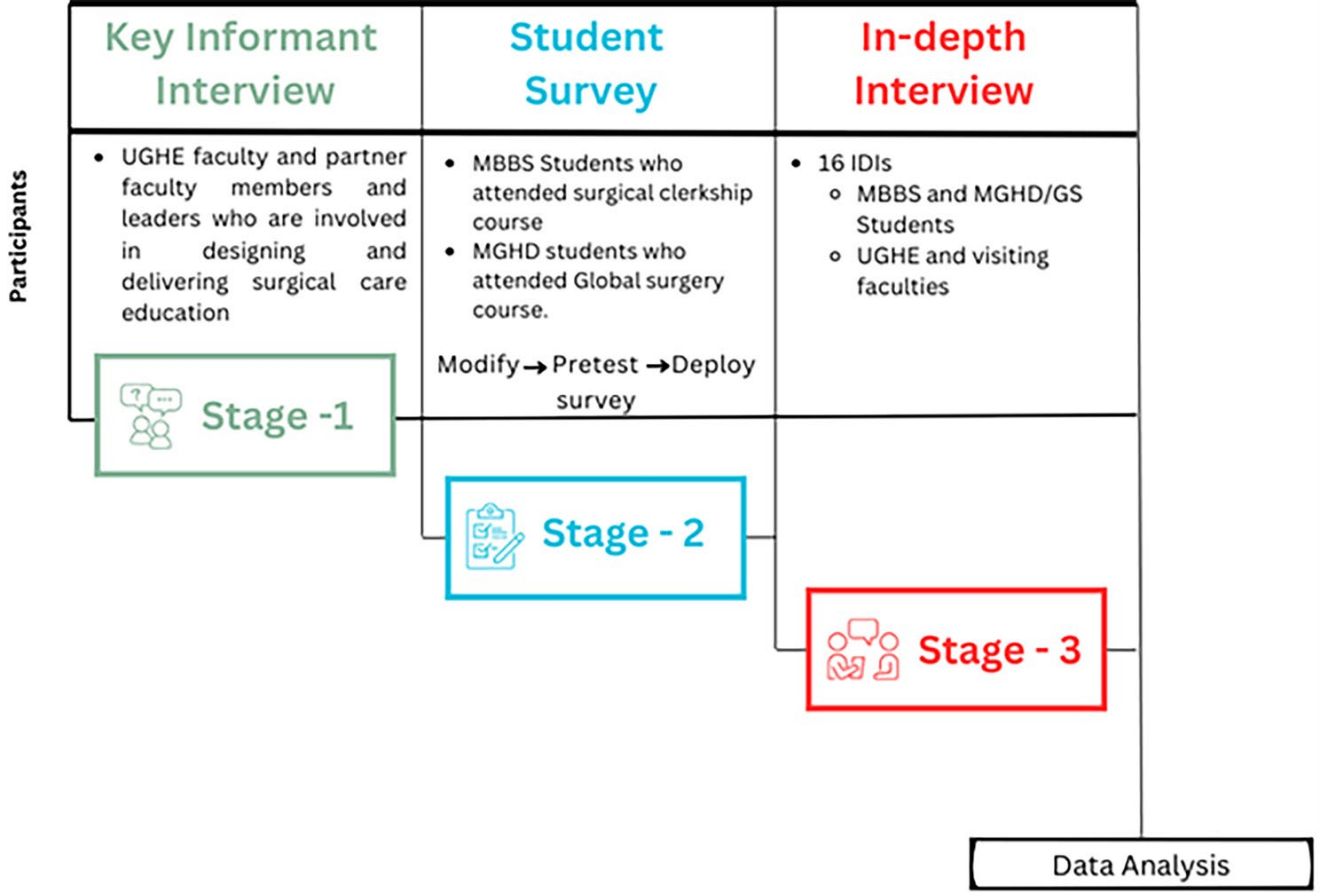
Methodologic approach for the sequential explanatory mixed methods design

This is a mixed methods study with three stages (Fig. 1). The study used a sequential exploratory mixed methods design. Results from qualitative key informant interviews (KIIs) in stage 1 were used to inform a quantitative student survey in stage 2, and we carried out methods level integration between these two. In depth interviews (IDIs) in stage 3 followed the student survey in a sequential explanatory mixed methods design. All the data was triangulated and we established and displayed data relationships and integrated in our interpretation and reporting. This study used qualitative and quantitative methods to collate the data merged in the interpretation phase to develop a comprehensive analysis of the partnership-based surgical education programs. The study participants included UGHE leadership, students, faculty, and partners who participated in surgical education programs at UGHE.

### Study setting

The study was implemented at UGHE; a private, not-for-profit, accredited health sciences university with its campus located in the rural North of Rwanda with a place-based education ethos [13] The university depends on local and global partnerships to provide rounded education and training, with visiting faculty from partner institutions contributing to teaching in person and online. The university delivers surgical education to its third- and fourth-year undergraduate medical students and Masters in Global Health Delivery (MGHD) students taking the Global Surgery option. The undergraduate medical course was started in 2019 with a six and half year program that includes a master’s program through the MGHD. The first clinical students started their clerkship class in 2021. The MGHD/ Global Surgery program (MGHD/GS) started in August 2023 [17]. Students are represented from across sub-Sharan African countries [18]. In 2024, there were 66 undergraduate and nine postgraduate students who took surgical education modules at UGHE. At the time of the study, the undergraduate surgical program had been running for 2 years with 66 students who had completed surgery (currently 108), while the MGHD Global Surgery had been running for 1 year with 9 students (currently 15).

Surgery modules at UGHE include the Junior Surgery Clerkship, the Senior Surgery Clerkship, Introduction to Global Surgery, Foundations of Global Surgery, Leadership in Global Surgery, Global Surgery Ethics, Partnerships, and Humanitarian Surgical Care, Global Surgery Policy and Advocacy, Quality Improvement and Innovations in Global Surgery, and Global Surgery Research Practicums. The modules comprise both clinical training, didactic components (flipped classroom, debates, student-led seminars, in-person and online classes, research seminars, case-based presentations), simulation-based training and gamification for surgical procedures including technical skills and non-technical skills, and community engagement sessions. Students in our study include both MGHD and Bachelor of Medicine; Bachelor of Surgery (MBBS) learners who received any form of surgical education at UGHE, all of which are partnership-based.

### Study design

The study’s inclusion criteria are students and fellows who are currently enrolled in or have completed, at least one surgical education module or course (didactic or clinical), at UGHE within the last three years. Faculty members from UGHE and its partner institutions, as well as external partners who had actively participated in UGHE’s surgical education programs during this period, were also considered eligible.

The exclusion criteria for this study were UGHE faculty members involved in any surgical education programs without partner involvement. Additionally, partnerships that have been initiated but have not yet been implemented were excluded to ensure that only active, completed partnerships were studied.

### Sampling method

Purposive sampling was used to recruit participants for the Key Informant Interviews (KII). The participants were selected based on their role in establishing partnership-based surgical courses at UGHE. These included leaders and faculty from both UGHE and national or international partner institutions. For the quantitative survey, we carried out a total sampling of all undergraduate and postgraduate learners who met the inclusion criteria. Eligible participants were contacted via email listservs. Sampling for the in-depth interview used a mix of purposive and snowball sampling to recruit participants who had experience in surgical education both from UGHE and partner institutions.

### Sample size

Qualitative interviews were conducted until thematic saturation was reached, and this determined the sample size. For total sampling on the quantitative electronic survey, 66 undergraduate students and seven postgraduate students (73 students in total) met the inclusion criteria.

### Data collection tools

A structured interview guide was used for both IDI and KII interviews. Two semi-structured KII guides were developed based on an international partnership framework- the Fair-Trade Learning framework with input from local global surgery context and content experts with expertise in partnerships for face and content validation [14]. Six authors including qualitative experts selected the interview guide questions and these were face validated and content. The information generated from the KIIs was then used to design and update the semi-structured IDI interview guide based on feedback from key informants on the partnership-based model.

The student survey tool was designed within an exploratory sequential framework, based on results from the qualitative interviews, built on the Fair Trade Learning framework. The survey questionnaire was also designed and modified based on information from the KIIs in an exploratory mixed methods manner. It was pre-tested with MGHD students who had not participated in surgical education programs and reviewed before it was deployed. It was administered through email using Microsoft Forms. There were no available internationally validated tools when the study was conducted. The student survey tool was divided into three sections. The first section provided a summary of the informed consent form that had been shared with the participants. The participants were asked if they had reviewed the consent form and were willing to participate in the survey. The second section collected relevant demographic information such as sex, year of medical education, and the type of surgical training in which the students were involved. The third section aimed to assess students’ satisfaction with the courses provided by partner institutions. A Likert scale was used to measure the level of agreement with specific statements related to partners’ engagement, communication, and achievement of learning outcomes using different teaching modalities.

### Ethics approval and informed consent

The study received ethical approval from the UGHE Institutional Review Board (reference number UGHE-IRB/2024/293). All participants involved in the survey, as well as those who took part in the interviews, provided written informed consent to participate.

### Reflexivity

The primary investigators are an Ethiopian male and a Kenyan female, who were at the time of the study, pursuing a master’s degree in global health delivery in global surgery. They are currently alumni and are not obliged to the institution. Other investigators are originally from Nigeria, Sierra Leone, and the United States, completing a diverse group. Approximately 30% of authors are female. Four authors were students, two were primarily external partners, one was a Global Surgery fellow, and three were surgery faculty. No potential conflicts of interest arose with respect to evaluating a program that one administrates or leads as the primary investigators kept a reflexivity journal throughout the study period.

### Data collection

After ethical approval and obtaining informed consent, we commenced data collection by conducting KIIs. All KIIs were conducted in English. Before each interview, the participants were taken through the informed consent and allowed to ask questions or seek clarification. Those who agreed to participate were asked to share a signed consent via email (for virtual interviews) or fill in the consent form (for in-person interviews). The two principal investigators took turns leading the interviews. When one served as the primary interviewer, the other would take notes and ensure the recording was done well. All recordings of the virtual sessions started with a recording of this consent process where verbal consent was used in lieu of physical. The principal investigators conducted all interviews, including virtual interviews, in a private, designated, and secure location. Online interviews will be held for those not physically present at the UGHE campus through a secure Microsoft Teams link. Interview guides were used for these sessions.

An online version of the survey tool was developed on Microsoft Forms based on KIIs. A unique survey link was generated and shared by email to all survey participants, and the information and consent form was attached. The first step of the survey provided a checkbox asking if the participant had reviewed the consent form and was willing to participate. An option for yes led to the survey, and those not providing consent were allowed to end the survey at that stage. Follow-up emails were shared to remind the participants to fill out the form. In addition, the principal investigators worked with the student representatives to share the survey link on their WhatsApp channel to enhance participation.

### Data cleaning preparation and analysis

The principal investigators reviewed all transcripts for accuracy, making minimal edits for clarity before renaming them with study identification numbers and uploading them to Dedoose qualitative analysis software for coding. A hybrid coding approach was employed, combining deductive codes based on the literature review and inductive codes from the independent analysis of KII transcripts [19]. After discussing emerging patterns, they refined the codebook and ensured intercoder agreement through daily check-ins. Thematic analysis led to the identification of four key themes. Excerpts were also evaluated using adapted Fair-Trade learning indicators for evidence of convergence (Please see Appendix 1). The primary investigators conducted participant interviews, maintained a reflexivity journal, and held debriefing sessions to ensure rigor. Findings were also triangulated across qualitative and quantitative data sets to enhance reliability and validity.

All survey responses were downloaded into Microsoft Excel. Missing or incomplete responses were carefully reviewed and excluded. A codebook was created to assign numerical codes to the Likert scale responses to facilitate their transfer to SPSS. The Excel files were then imported into IBM SPSS Statistics 20 for analysis, and the codebook was integrated into the variable view. The data was summarized using descriptive statistics, which included calculating the percentage of responses for each categorical variable.

### Data management

All study data collected from the interviews and online survey were stored securely and access to the study materials was limited to the principal investigators. Interview transcripts were anonymized by using unique identifiers, to ensure confidentiality and anonymity. Both principal investigators maintained a password-protected folder of all research data as backup storage. Data from the study will be kept in UGHE’s academic office for safekeeping and destroyed after ten years.

## Results

### Qualitative result

A review of institutional documents conducted prior to interviews shows that there are a total of 34 individuals (at the time of data collection), from 24 different institutions, who are involved in surgical education as a partner or direct UGHE faculty of which six are UGHE faculty. We conducted 21 interviews, five of which were KIIs. These key participants were individuals from UGHE and its partner organizations with extensive experience in partnership-based surgical education and were involved in establishing surgical education at UGHE. Two KII participants were UGHE faculty members (40%), three were experts from partner organizations (60%), with one individual from a local Rwandan institution and two from international partners. Furthermore, we carried out 16 in-depth interviews, with six students (37.5%), three UGHE faculty members (18.5%), and seven (43.5%) from UGHE partner institutions involved in delivering surgical care education, three of which were local within Rwanda, and four were international institutions based in High-Income Countries Table 1.

**Table 1:** Demographics of qualitative interview participants

Sociodemographic Characteristics (*N* = 21)		Number (Percent)
**Sex**		
	Male	18 (86%)
	Female	3 (14%)
**Level of education**		
	Undergraduate student	4 (19%)
	Postgraduate student	2 (10%)
	Graduate (Bachelors)	1 (5%)
	Graduate (Masters/Fellowship)	14 (67%)
**Affiliation**		
	Individual	2 (10%)
	University	14 (67%)
	Non-governmental organization	1 (5%)
	Teaching Hospital	4 (19%)

### Theme 1: a needs-based approach is used to determine the suitability of forming partnerships

Key informants were asked to describe the process of forming partnerships and the rationale for adopting a partnership-based model for the delivery of surgical education.

A participant defined partnership as

“*It’s a win-win connection between external players and internal players*,* in which there is really no giver-receiver. It’s an exchange.”* (Faculty 1, Rwanda).

Informants mentioned that the university conducted a Delphi study that gathered expert opinions from all around the world to design the curriculum for undergraduate surgical clerkship programs. The findings from this study led to partnership formation to support content delivery. This is better described by one of our informants who stated the following.

*“First*,* you should have an objective. Why do you need a partnership? That’s the very first thing we define at UGHE. Why do we need a surgical education partner? To do what? To develop this module? To teach this module? To come and help us teach this module? To do this research? To apply for this grant?”* (Faculty 4, Rwanda).

Informants identified common goals between the two partners that laid down the groundwork for further collaboration. These common goals include training an additional surgical workforce, alleviating the surgical burden of diseases such as cancer through patient care, and pushing surgical academic excellence through research.

*“…And we’re going to be talking about three specific areas that we would think we will collaborate on in the future. One is cancer*,* global cancer treatment and control. The second is on climate change and global health. And the third is on community participatory research…”* (Partner 2, USA).

Another participant explicitly mentioned how UGHE is helping its partners in research and community engagement.

“… *we have developed research and right now we have I think like 10 top topics that we have submitted to UGHE they are helping us to design research…”* (Partner 3, Rwanda).

In many interviews, informants mentioned several qualities that make UGHE a desirable surgical education partner. Most notably, UGHE deliberately reaches out to partners and maintains respectful relationships. The sub-theme of maintaining “respectful relationships” is explicitly mentioned throughout the interviews, appearing 15 times across four different interviews. While one instance carried a negative connotation - “unnecessary respect”—the majority were positive, with “mutual respect” being the most frequently mentioned. When practically elaborated or contextualized informants referred to respect manifesting in trust, reciprocity, shared expertise, and appreciation. The following quote illustrates how respect between partners played a role in building trust between relationships:

*“…. it’s a trust that’s built [on] going for a common goal and having a mutual respect and an appreciation for what everybody brings to the table that gives us a chance to have some substantive discussion.”* (Partner 4, USA).

Participants also highlighted qualities such as the university’s approach to education, equity, mentorship, and innovation to address pressing global health issues. Additionally, participants identified leadership qualities in UGHE that aim to excel in educational excellence, focus on the bigger picture, and uphold a strong vision:

*“What is so different about UGHE is*,* first*,* that it is new. This is a very new university. And I think the opportunity to start fresh and to build a university based on the idea of health equity is extraordinary. I [also] think [it’s] leadership is visionary. People are really thinking ahead*,* looking at the big picture.”* (Partner 2, USA).

The results with both UGHE faculty and its partners show that the model of surgical education highly relies on two-way communication between individuals from the partnering institutions. This communication serves as a tool used to ensure bilateral cooperation exists and mutual benefits are achieved among partners. Communications are often mentioned as meetings and regular updates on projects between responsible individuals from partner institutions.

### Theme 2: UGHE and its partners worked towards equitable outcomes

UGHE has centered equity on how it works, including creating and nurturing partnerships. The participants shared the value of a synergistic relationship where all partners play an active role in the partnership. UGHE offers a research and development platform that is strategically integrated within the community. The university also provides its partners with valuable opportunities for community engagement, featuring practical, hands-on programs designed to bring meaningful healthcare solutions.

*“There is always a give and take in partnerships. If it’s just a take*,* [and] take*,* [and] take*,* then you’re a recipient. It’s a donation. There is no equity there.”* (Faculty 4, Rwanda).

When asked if the partnerships they are engaged with at UGHE are equitable, the participants gave mixed responses. The majority affirmed that the existing model promotes equity, but more work remains to be done. Participants also mentioned the need to share resources and the value of good leadership as key components of driving equity:

*“I think that the way we’re doing the system that’s in place doesn’t allow for such an imbalance in the partnership… they’re carefully selecting the partners. And [the] partnership is also working very well… nobody comes thinking that they’re giving you something. They know that they’re partners. They know that they have something to gain from this partnership as well.”* (Faculty 1, Rwanda).

Similarly, another participant shared the following perspective on how UGHE and their institution aim to address equity.

*“…. regardless of any topic in global health*,*… equity challenges are always going to be present. That’s just part and parcel of the material and the dynamic. I think ways that we practically address this is we acknowledge them up front.” (Partner USA)*.

Despite the efforts made, it was clear that more could be done to ensure a bidirectional flow of benefits. Ensuring equal opportunities for individuals on both sides of the partnership is crucial. Respondents mentioned the need to have equal opportunity for travel that can potentially benefit faculty at UGHE and partner hospitals. This will further enhance knowledge transfer and equip local staff with necessary skills such as minimally invasive surgery techniques that are not practiced in Rwanda’s settings.

### Theme 3: positive outcomes of the partnership model for students and the institution

Most respondents who were UGHE faculty mentioned that one of the most important advantages of this partnership-based approach is the opportunity to expose medical students to international standard medical education. Others pointed out the access to state-of-the-art learning platforms such as Canvas, Entrust, E-Libraries, and simulation materials, which greatly enhanced the learning experience. One of our key informants said:

*“…We get the best of training for our students. That is [of] international standard by partnerships. We get great ideas about medical education. Because medical education is not just education like there are techniques to these things…”* (Faculty 3, Rwanda).

The diversity of exposure was not limited to the content of the classes they were taking. Most students mentioned having the chance to interact with professionals abroad helped their global understanding of healthcare. This experience is amplified for students who participated in master’s courses and Global surgery advocacy fellowship programs that are facilitated by UGHE in partnership with several international institutions. Such programs created a platform for students to work and interact with other students who participate in these programs from abroad. One participant mentioned the following.

*“…And for the fellowship as well*,* I had a chance to collaborate with other experts based in LMIC*,* including people from Latin America*,* from West Africa*,* which I wouldn’t be able to get to work with…”.* (Student 7, Rwanda)

Another crucial benefit mentioned in the interviews was the inspiration the students in master’s programs derived from their exposure to various visiting faculty members who had made significant accomplishments in global surgery. Undergraduate medical students also noted that most of the faculty members were “kind” in their approach and were able to influence the students through their work, accomplishments, and contributions to the field.

*“… It has influenced me so much. And it has somehow shaped my mindset as well… So… the more you take that course*,* the more you’re inspired or the more you are motivated to take on further steps in the field….”* (Student 7, Rwanda).

### Institutional advantages

Several advantages were mentioned that benefited both UGHE and its partners on an institutional level. UGHE’s partnership promoted regular morning meetings and enhanced research efforts in partner organizations. These included the creation of a more diverse curriculum, the opportunity to gain diverse experience in surgical care education and research, and leadership incubation that benefited both sides. One of the participants summarized this as follows.

*“… [through partnerships we can] develop and strengthen both faculties*,* create new curricula*,* train more students*,* work on research collaboratively*,* which hopefully then creates*,* routes for independent collaborative research funding…”* (Partner 10, USA).

Partnerships facilitated resource sharing between institutions. It was also mentioned that faculty at UGHE gained experience in advanced teaching skills, which helped their students and enriched them as professionals. The partnership also increased the workforce, helping UGHE train professionals in different sectors. Other benefits included research management, grant writing, and the opportunity to leverage technology to advance medical teaching through simulation and interactive platforms such as Entrust. One of our key participants mentioned the following:

*“…it helps us save resources. We can’t afford to hire 12 full-time faculty to teach global surgery or 12 full-time faculty to teach in the basic sciences. The presence of these partnerships helps us save a lot of resources…”* (Faculty 4, Rwanda).

Partners have supported UGHE in building its faculty capacity, and with time, the internal faculty is ready to take on more responsibilities in the leadership of the institution. As one of our key participants put it, *“The goal is to develop partnerships that eventually allow low—and middle-income country institutions to really stand on their own.”* Participants indicated that UGHE is on the path to sustaining the benefits gained from its partnerships indicating that there is a clear exit strategy and a path for its partnerships to evolve.

*“We have appointed deputies. These deputies will hopefully become full department heads next year. That does not mean the partnership ends. It changes from leadership to mentorship. To advocacy. To distant support.”* (Faculty 4, Rwanda).

### Theme 4: challenges faced in delivering surgical education using a partnership model and proposed solutions

These themes capture participants’ feedback that highlights the challenges faced in delivering surgical education using a partnership model. There are 3 subthemes included under each team. The first one is Partnerships and Operations related, and the second one is Educational Challenges Linked to Service Delivery. The last theme will present the proposed solutions for the challenges identified. Each will be discussed below.

### Partnership and operational challenges

Participants identified several challenges and operational difficulties that are related directly to the partnerships. Participants mentioned “logistics hurdles”, associated particularly with visiting faculty who need visas, transportation, and accommodation in Rwanda. Participants highlighted contextual differences due to varied operational settings that sometimes led to culture shock and confusion for undergraduate students. Moreover, interviews also revealed occasional gaps in communication between the university and the hospital staff or visiting faculty. These included double booking of schedules, and incomplete documentation and transmission of student progress among teachers, such that one visiting surgeon/clinician was unsure of the extent of previous training. A student in one of the interviews said the following:

*So*,* I used to request* [the] *surgeon*,* I want to assist on this on [Procedures]*,* but they did not trust that I had the skills to… to assist him or her very well…. I think that the reason is they’re not aware of the training we have had prior.”* (Student 4, Rwanda).

The impact of operational and cultural variation between institutions is best described in the following participant’s quotation:

*“So*,* the students will sometimes get confused*,* because when they’re with the local faculty*,* they’re asked…. take a detailed history*,* take a physical exam*,* and so on and so forth. And then there is a visiting faculty*,*… go straight to the CT finding*,* MRI finding.”* (Faculty 1, Rwanda).

### Educational challenges linked to service delivery

Feedback from the interviews identified supply chain shortages and equipment breakdowns, that led to the cancellation or delay of surgical procedures. This directly impacted the clinical training for medical students undertaking time-bound surgical education. Additionally, responses showed that for the local training sites, if the internal faculty engaged in serving as clinical preceptors were not available or busy, the students ended up getting inadequate supervision. This is reflected in both student and faculty interviews:

*“We have faced challenges because we were alone on the ward or in the OR*,* or we were just dealing with the local*,* healthcare providers who don’t necessarily understand what our objectives are… that has impacted us in a negative way.”* (Student 6, Rwanda).

Another challenge mentioned by a student was the time constraint in going through their materials and grasping the concept of their lessons. One participant mentioned the following:

*“…time was [a] constraint because I feel some of the modules were so compacted*,*… I feel what could be done best always if we are able to expand this time so the students take it little by little so they can really*,* really absorb the good content that they are given…”* (Student 6, Rwanda).

### Proposed solutions

The participants provided feedback on interventions to improve this model to achieve maximum benefit for all involved. One of the recurring themes was the need to create a formal mechanism for feedback:

*“Not only the one we write and just evaluate different faculty*,* but it should also have a genuine feedback*,* one-on-one feedback at a certain point with maybe the head of the department*,* so we can give our genuine opinion about it.”* (Student 2, Rwanda).

A participant from one of the hospitals that hosted UGHE students during their surgical rotation called for better integration between the hospital and the university to further strengthen their role as a teaching hospital:

*“Try to plan together as one unit*,* meaning that all the faculty in the UGHE*,* in the university are also having their protected time in clinical practice. So that’s the only way things can improve and both the university and the hospital benefit from that*,* I would say partnership*,* but by extension the [Name] hospital should be the university teaching hospital.”* (Partner 6, Rwanda).

### Quantitative findings

Online survey was sent to the undergraduate and postgraduate students who met the inclusion criteria. The principal investigators were exempted from this survey. This brought the target number of participants to 73. We had 31 responses from the survey, bringing our response rate to 42%. One response was discarded on account of incompleteness, and others invited to the survey did not respond. The MBBS students reported participating in classroom lectures, skills workshops, simulations, and clinical training sessions facilitated by visiting faculty. The MGHD students participated in classroom lectures and webinars facilitated by visiting faculty Table 2.

**Table 2:** Demographics of respondents to the quantitative student survey

Sociodemographic Characteristics		Number (Percent)
**Sex**		
	Male	14 (46.7%)
	Female	16 (53.3%)
**Level of surgical education**		
	4th-year MBBS students	15 (50.0%)
	5th-year MBBS students	11 (36.7%)
	Masters in Global Health Delivery - Global Surgery students	4 (13.3%)

### Respondents’ perception of sessions facilitated by the partner faculty

**Fig. 2:**
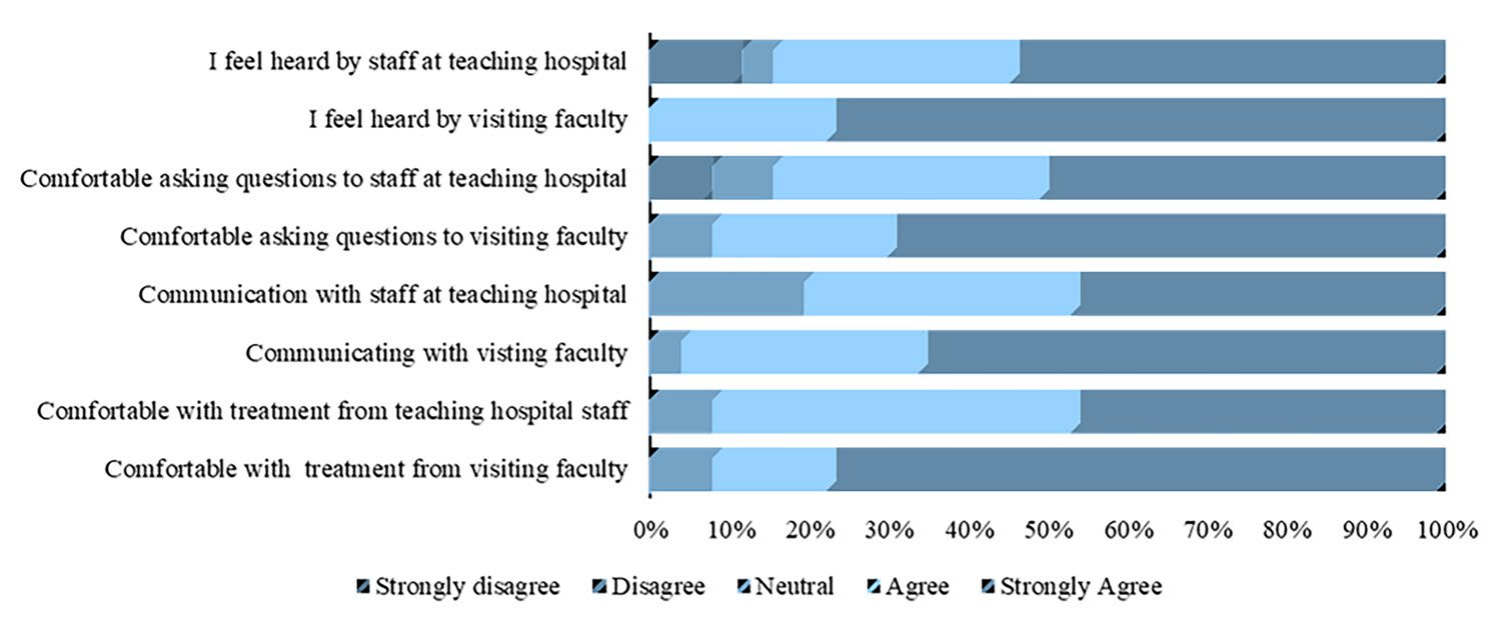
Bachelor of Medicine, Bachelor of Surgery (undergraduate) student perceptions on interaction with partnering faculty

**Fig. 3:**
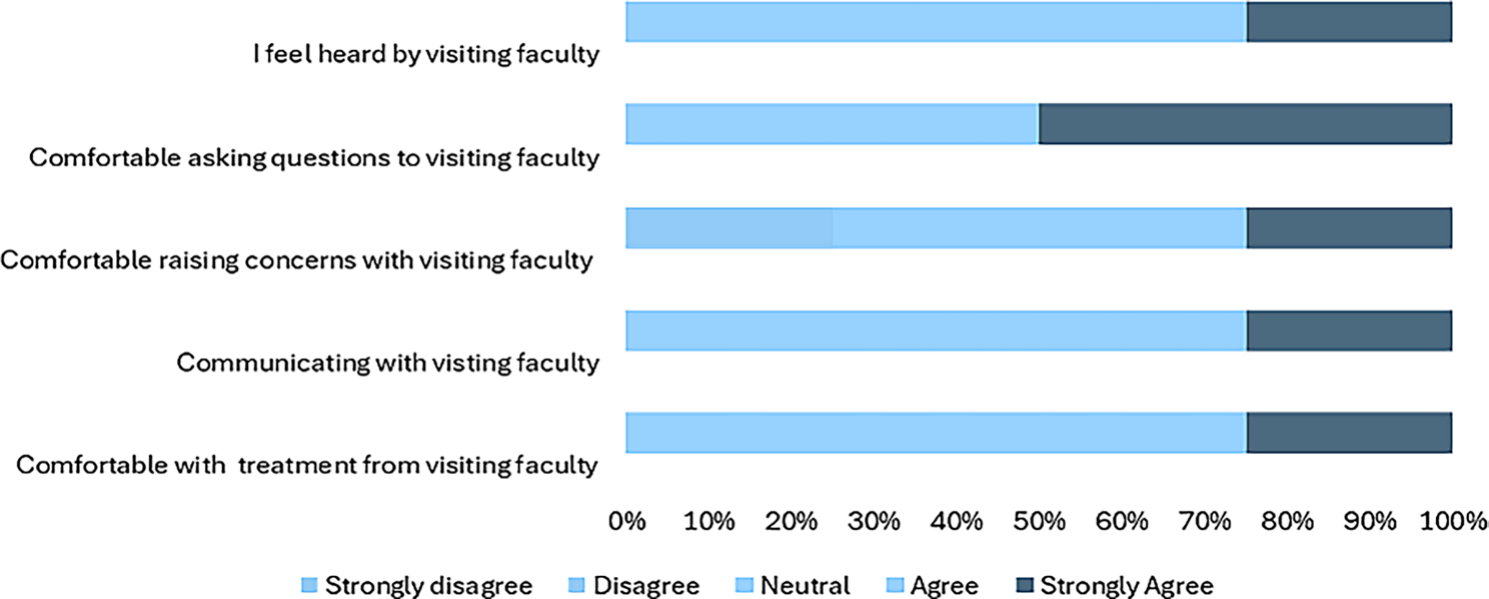
Masters in Global Health Delivery (postgraduate) student perceptions on interaction with partnering faculty

**Fig. 4:**
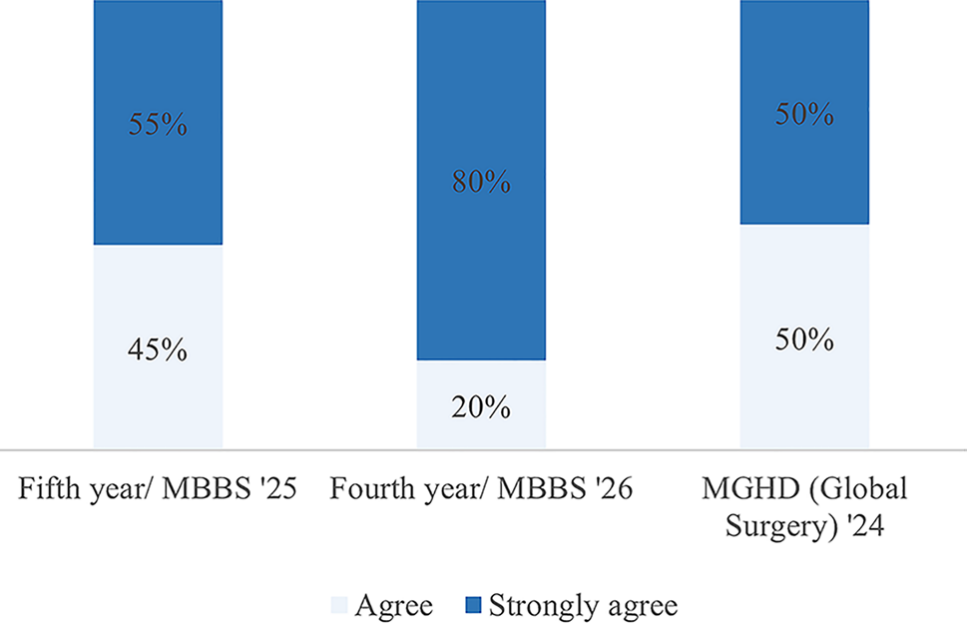
Response to “Didactic surgical education sessions aided my understanding and engagement.”

**Fig. 5:**
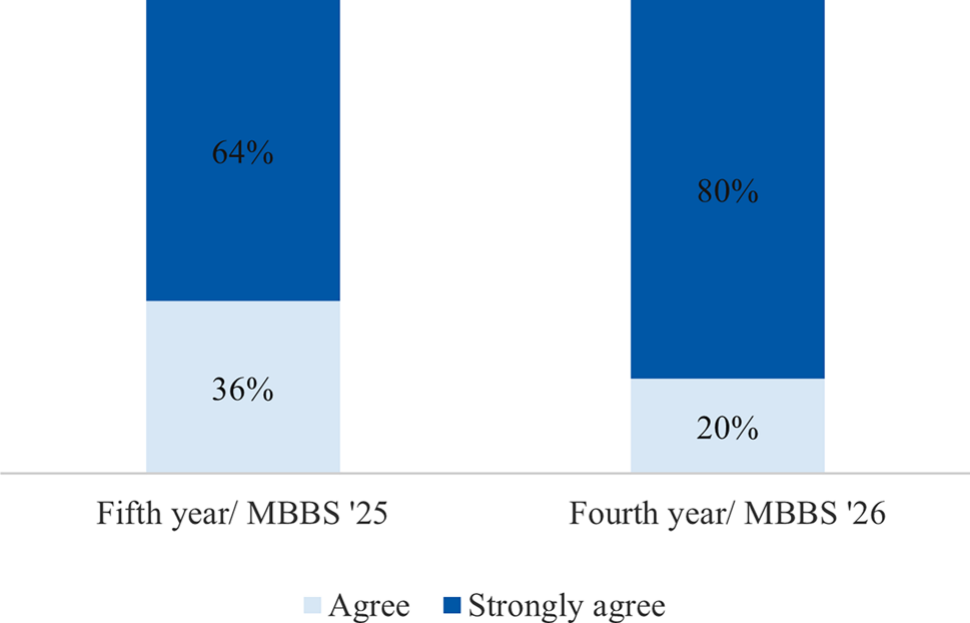
Response to “Simulation sessions aided my understanding and engagement”

The respondents had a positive response to the sessions facilitated by the visiting faculty. The survey asked for their level of agreement with the statements that described their interaction with partnering visiting faculty (Figs. 2 and 3). While the satisfaction level varied within student groups, the differences were not significant. Student perceptions about partnership driven didactic sessions and simulations are as shown (Figs. 4 and 5). The students were also asked if they felt that they had adequate time for the sessions facilitated by visiting faculty. Compared to the undergraduate students agreed overwhelmingly with this statement, 50% of the postgraduate student respondents did not feel they had adequate time with visiting faculty, resulting in a statistically significant difference (*p* <.001).

The quantitative data supports the student’s perception of the interaction with partner faculty from both international and local institutions. 93% (*n* = 27) of the participants reported a favorable interaction with the international faculty. They agreed with the statements that they were comfortable with their treatment by the visiting faculty, felt comfortable engaging and asking questions, and felt heard by the visiting faculty, but 7.7% reported a neutral response to these statements.

Regarding interaction with the partners at the teaching hospitals, many undergraduate students responded positively that they were comfortable asking questions and felt heard by the staff at the teaching hospital. Up to 3.8% and 11.5% of the undergraduate participants indicated that they were uncomfortable asking staff questions at the teaching hospital and did not feel heard by the teaching hospital staff respectively.

### Perception of interactions with partner faculty

The students were also asked about their level of agreement based on their interaction with partner faculty from both international and local institutions. The local institutions referred to partner teaching hospitals where the bulk of the clinical teaching takes place. The students were also asked if they felt their learning improved due to these interactions.

93% (*n* = 27) of the respondents reported a favorable interaction with the international faculty. They agreed with the statements that they were comfortable with the treatment from the visiting faculty, felt comfortable engaging in and asking questions, and felt heard by the visiting faculty. 7% reported a neutral response to these statements. There were no significant differences in their responses.

When it came to interaction with the partners at the teaching hospitals, the majority of the undergraduate students responded positively that they were comfortable asking questions and felt heard by the staff at the teaching hospital. 8% and 11.5% of the respondents disagreed with the statements that they were comfortable asking questions to the staff at the teaching hospital and felt heard by the teaching hospital staff respectively Table 3.

**Table 3:** Joint display: combined qualitative and quantitative findings with study objectives

	Parameters	Qualitative findings (Representative quotations)	Qunatitative findings (Online survey)					Meta-inference
Objective 1: Effectiveness of UGHE’s partnership-based models in achieving educational goals.	What were the benefits of partnership-based model of surgical education?	*We get the best training for our students. That is [the] international standard by partnerships. We get great ideas about medical education. Because medical education is not just education*,* like there are techniques to these things…” (Faculty 3*,* Rwanda)*	* 80.8% of Students from MBBS “Strongly agree” that visiting faculty improved their overall experience, and 19.2% of them “agree.” * MGHD 75% “strongly agree” 35% “agree”					The partnership-based model significantly enhanced training quality by exposing students to international standards and innovative techniques. Quantitative data supports that visiting faculty improved student’s educational experience.
	How effective is the partnership-based model	*Entrust [Educational platform] cases I really did like them and they were facilitated by different people depending on the case we have if it is an orthopedic case we had an orthopedic from Kabgayi help with that…I liked them [cases on the platform] because they were more interactive… more than just having like a lecture” (Student*,* Rwanda)*	Student survey	MBBS		MGHD		The partnership-based model demonstrates high effectiveness, as students appreciated its interactive, case-based approach tailored to specific disciplines, enhancing engagement beyond traditional lectures. Survey results further highlight this with strong approval for clinical and practical teaching.
				Strongly agree (%)	Agree (%)	Strongly agree (%)	Agree (%)	
			Didactic teaching	69.2	30.8	50	50	
			Clinical/ Practical	73.1	26.6	N/A	N/A	
			course material understanding	57.7	38.5	25	75	
	Has equity been successfully integrated into UGHE’s partnerships?	*“I think you just have to be intentional about it…[and] think about it at every step….You would hope…[that] the more you do it*,* the more you practice it*,* the more it becomes just the natural way that you do things.” (Partner 10*,* USA)*	N/A					The study found evidence of genuine efforts to set up systems and structures that promote equity.
Objective 2: Identify successful partnership strategies, ethical challenges, and best practices in UGHE’s collaborative surgical education	How and why the partnership-based model was formed?	*“We can’t afford to hire 12 full-time faculty to teach global surgery or 12 full-time faculty to teach in the basic sciences. The presence of these partnerships helped us save a lot of resources”* (*Faculty 4*,* Rwanda)*	N/A					UGHE adopted the partnership-based model to fill in critical gaps as part of its strategic growth.
	what were perception of UGHE as a surgical education partner?	“*UGHE has an incredible strength in education*,* incredible commitment to equity*,* incredible mentors*,* and a willingness to invest in things that are innovative” (Partner 3*,* USA)*	N/A					UGHE has built a reputation as a reliable, trustworthy and credible academic partner.
	What challenges does this model face?	*“Due to other responsibilities of hospital management that I’m involved in. Sometimes it becomes difficult to really give all that I want to give to them [ the studnets in terms of teaching].” (Partner 6*,* Rwanda)*	15.4% of the MBBS student surveyed responded that they either felt ‘neutral’ or ‘disagree’ with the statement “I am comfortable asking questions to staff at teaching hospitals”					The 2022 Junior Surgical Clerkship Assessment survey documented similar challenges identified in this study.
			11.5% of the students did not feel heard by staff at the teaching hospitals					
	Does the partnership-based model promote equity?	*“I can testify that the equity is there…. everyone is benefiting…. the faculty at UGHE*,* they come and cover some clinical activities. We learn from them. They learn from us. We work as a team. On my side*,* I am very happy with the partnership.” (Partner 7*,* Rwanda)*	N/A					UGHE’S partnership model promotes equity for its students, faculty and partners. There is, however, room for improvement.
	How can the partnership-based model be improved?	“*Not only the one we write and just evaluate different faculty [Post course evaluation surveys]*,* its should have a genuine feedback*,* one-on-one feedback at a certain point with maybe the head of department*,* so we can give our genuine opinion about it.” (Student 2*,* Rwanda)*	*The model is transformative*,* but some visiting faculty should be closely monitored to avoid introducing individual motives into the model. This way*,* it can stand the test of time*. (Anonymous, MGHD)					A formal feedback structure needs to be set up to collate both quantitative and qualitative feedback from all stakeholders.

## Discussion

Our research evaluated UGHE’s partnership-based model for surgical education in Rwanda. The mixed-methods study shows that UGHE’s needs-based approach aligns with shared objectives, providing students with diverse exposure supported by technology. The results indicate that UGHE maintains partnerships that are mutually beneficial, allowing partners to engage in research and community programs. However, the model faces institutional and operational challenges. Addressing these challenges will help UGHE and similar institutions enhance modern medical education.

To define success in partnership, it is essential to examine the origins of the relationships, the reasons for their formation, the common goals set by the partnering institutions, and the expected outcomes [11]. It is therefore crucial to start with UGHE’s intention to form these partnerships. This study identified that UGHE followed a needs-based approach to identify gaps and define objectives for its partnerships. Literature by Alemu et al. [8, 20] highlighted the need for partnership to support faculty and curriculum development and address funding shortfalls. Similar evaluations of surgical education programs in Ethiopia and Uganda showed how partnerships filled the critical gaps in experienced faculty and financing [9, 16]. UGHE has utilized this model effectively to meet the gaps and provide its students with diverse exposure to experienced global surgery specialists.

Peluso et al. recommended an assessment of partnership outcomes [10]. Both students and faculty reported individual and institutional benefits of the partnership-based surgical education model. Diverse exposure to different specialties and world-class experts was a clear benefit for not only the students but also the faculty who got to build their medical education capacity through formal and informal training by the external faculty. These results are similar to the findings of a study done in Uganda which called for partnerships that build on sharing resources, including Information and Communication Technologies and libraries [16]. UGHE’s partnerships have allowed students to access virtual libraries from well-resourced universities based in the Global North and to access learning platforms like Canva and ENTRUST gamification [21]. This, along with the development of both the physical infrastructure and workforce to deliver simulation training, has enhanced learning for students who reported that they enjoyed the interactive discussions more than the lectures. UGHE has also grown in research, grant writing, and training capacity.

This benefit has often been bilateral, and UGHE partners have shared how their institutions have gained from this partnership model. UGHE’s partnership encouraged regular morning meetings and improved research efforts in partner hospitals. UGHE’s partners also benefit from UGHE’s ideal position in Global Health and Equity. The institution has been an ideal partner for practical rural community engagement programs. UGHE contributes to its partnership by facilitating such community engagement programs and by enabling research and health promotion efforts in these underserved regions. The university also supports teaching and training efforts for this partner through its platforms. Mutual benefits are fundamental to ensuring equality in partnerships and to sustain collaborations [14] Similar studies indicated bilaterality in the programs studied. For instance, a study done on the partnership model adopted in Uganda’s Makerere University with Yale showed that the program had impacted both institutions [22]. UGHE is intentional in the bilaterality of engagements and offers a conducive environment to find inclusive solutions to global healthcare challenges.

Institutional partnerships are often complex and ensuring a successful partnership has always been challenging. The challenges sought in this partnership model were mainly classified into institutional and systemic challenges. This included logistics, cultural differences, practice variations, miscommunications, and lack of funding and resources to facilitate the training sessions. These challenges have frequently been mentioned in literature and are not novel in UGHE’s partnership. For instance, funding has been mentioned as one of the common reasons why academic partnerships fail to continue [23]. The challenges of logistics, such as transport, accommodation, and visas, have also been mentioned in Citrine et al. as hurdles in the smooth operation of Nepal’s partnership with international institutions [24]. Such challenges can be mitigated by proper planning, accounting for cultural differences, and addressing visiting faculty expectations.

Another challenge frequently mentioned by faculty and students is the difference in medical practice between well-resourced Western institutions and limited-resource settings. Bodnar et al. discussed the challenge of skills applicability, where fellows trained in well-resourced settings find it difficult to apply their new skills in their practice due to a lack of resources, adaptation, or manpower [22]. UGHE conducted a Delphi study to address these challenges and identified topics to be taught in its surgical courses which are used as a guiding framework for visiting faculty [25]. Additionally, for the transfer of knowledge from visiting faculty to students to be effective, it is essential to have adequate material and infrastructure that facilitate the teaching and learning experience. As noted by Almeida *et. a l*, the availability of resources plays a crucial role in knowledge transfer [26]. Without the necessary resources, such as an adequate supply of gloves and drapes and functional equipment like autoclaves, the smooth operation of learning activities in teaching hospitals can be hindered.

August *et al.’*s definition of global health equity places partnerships at the core of the concept [27]. The paper asserts that mutually beneficial and power-balanced partnerships are essential steppingstones toward achieving global health equity. UGHE has actively engaged in a variety of academic partnerships, all focused on the common global goal of training healthcare professionals. Through these concerted efforts, UGHE and its partners have successfully trained future healthcare leaders equipped to tackle healthcare challenges in underserved regions. Additionally, these partnerships have facilitated research initiatives and community engagement programs, resulting in real solutions that positively impact these communities.

### Study limitations

The study was conducted at a single institution in Rwanda and its partner organizations, which limited the sample size and generalizability of the research. We also acknowledge that the survey response rate in this highly specific population of undergraduate and graduate students could have been improved. This was perhaps due to the high pressure of academic work competing with responses. However, the principal investigators conducted numerous interviews with stakeholders with diverse experiences with other institutions to enrich findings. The study’s findings provide valuable insights into the establishment of global academic partnerships for medical education and support similar efforts aimed at addressing the medical education gap in Sub-Saharan Africa.

Most of the participants in the online survey are medical students training at UGHE with full scholarships offered by the university and its partners. This may have introduced a bias in the students’ responses. The investigators attempted to reduce this by anonymizing the online survey. They additionally conducted interviews with select representatives of the student population to identify any convergence between the qualitative and quantitative findings. In addition, while the surveys responses showed equitable distribution by sex (53.3% female), participation in the qualitative interviews were predominantly by male participants because of the purposive and snowball sampling used to recruit participants. Equity and better generalizability of study results would have been enhanced by purposive selection based on sex.

Lastly, this study focuses on stakeholders’ perceptions of the benefits and challenges of UGHE’s partnership-based surgical education model at both individual and program levels. It does not provide a direct evaluation of the program’s impact on workforce development or surgical service delivery outcomes for the community as the education model is still in it’s early implementation phase. As a way forward, we propose a future re-evaluation of the program and its impact on workforce expansion, surgical care, and medical education. Perspectives from individuals outside the narrow educational system, particularly from stakeholders that are not related to the university, would also be helpful for more in-depth evaluation, and this is being planned for further studies.

In conclusion, our assessment of the partnership-based model at UGHE found that it was a useful approach to providing benefits for both students and faculty, including a world-class surgical education program. There is room to amplify the benefits of this approach by addressing the gaps identified within the systems and institutions supporting medical education programs.

## Electronic supplementary material

Below is the link to the electronic supplementary material.

Supplementary Material 1

## Data Availability

The datasets used and/or analyzed during the current study are available from the corresponding author on reasonable request.

## Abbreviations

IDI: In-Depth Interview(s)
KII: Key Informant Interview(s)
LMIC: Low- and Middle-Income Countries
MBBS: Bachelor of Medicine, Bachelor of Surgery
MGHD: Masters in Global Health Delivery
UGHE: University of Global Health Equity
