# Supplementary material for: Assessing a partnership-based model of surgical education in the Global South: a mixed methods study of the University of Global Health Equity, Rwanda
Source: BMC Surg. 2025 Jul 3;25:258. doi: 10.1186/s12893-025-02996-5 (PMC12224651; doi:10.1186/s12893-025-02996-5)
Supplement: Supplementary file 1 — Supplementary Material 1 [file 12893_2025_2996_MOESM1_ESM.docx]

Appendices

1. Fair-Trade Learning Indicator Analysis

The principal investigators analyzed the qualitative data against adapted Fair-Trade Learning Evaluation indicators defined in Appendix 2. Table 6 shows the summary of this analysis.

| **INDICATOR** | **REPRESENTATIVE QUOTE** | **META INFERENCE** |
| --- | --- | --- |
| Common purpose | *we have done this as well, will establish a vision, a mission, a value statement that we can sort of go back to* (Partner 10, USA) | UGHE and its partners share a long-term goal of improving access and delivery of quality surgical care. |
| Host community program leadership | *“Partnership is not just with the global north, it's south- south, it's within Rwanda, it's within academic institutions in Rwanda, it's with the community in Butaro, it's also with Harvard, with Stanford. So everything we do at UGHE is partnership based....”* (Faculty 4, Rwanda) | UGHE is committed to diverse community engagement for its global surgery efforts. |
| Rights of the most vulnerable | *“… the same people who are always left behind by a system whose needs are not prioritized, the poorest, the most remote, the most vulnerable…. So this is why we started to feel that we should really put some emphasis into advocacy…advocacy to also engage in policies and policy development and policy research and policy implementation.”* (Partner 3, USA) | UGHE’s partnership is driven by local needs that address the inequities in surgical care. |
| Theory of change (student) | *“They benefited a lot from our expertise and we've benefited from them. So, it has been a wonderful experience for students to work with different mentors, different trainers and also seeing a lot of patients.”* (Partner 9, Rwanda) | UGHE partnership-based model has curated a conducive learning environment for all stakeholders with maximum student benefits. |
| Communication | “*the fact that the relationships do exist makes communication easy…And those communication channels are open and regular, which helps to avoid some of the conflicts that might otherwise arise*.” (Partner 8, USA) | Open and honest communication forms the basis of maintaining partnerships, addressing challenges, and adopting efforts based on reality. |
| Clarity of commitment and evaluation of partnership success | *“So even the partnership itself is feeding into the concept of equity. I think even making, fostering partnerships is because we want there to be some diffusion from places where resources may be more, teaching may be more, capacity may be more, to places where it's less, to bring them up to a point where everybody's looking at the same thing”* (Faculty 3, Rwanda) | UGHE’s stakeholders demonstrated commitment to shared goals and the achievement of mutual benefits. There is more work to be done to improve the evaluation process. |
| Transparency | *“...You are transparent about the budgets. Some people will not tell you the amount of money that they will collect on your head. We are transparent about budgets.* (KII 001, Senior Leadership) | UGHE’s growing and diverse partnership is a demonstration of its reputation as a trustworthy, transparent, and reliable partner |
| Partnership not program | *“Partnerships should not end up in dependency. If I, for the coming 10 years, depend on the University of Toronto to teach global surgery human resources, then it is a huge mistake. … the graduates, will learn how to teach it. And you will come back to teach it. That is the way of sustainability…. They need to evolve.”* (KII 003, Senior Leadership, Rwanda) | UGHE takes time to learn its partners before engaging in formal partnership as a means of ensuring long-term commitment to common goals |

Appendix 2: Definition of Fair-Trade Learning Indicators

| **INDICATOR** | **DEFINITION** |
| --- | --- |
| Common purpose | Mutually established long-term goals. |
| Host community program leadership | Community members have teaching and leadership roles, research efforts are community-driven and co-owned. |
| Rights of the most vulnerable | Vulnerable populations are recognized, and appropriate processes are in place to protect their rights. |
| Theory of change (students) | Partnerships motivations for student outcomes are identified and welcomed by diverse community stakeholders. |
| Communication | Clear lines of communication are identified, and communication is ongoing throughout the year. |
| Clarity of commitment and evaluation of partnership success | Ongoing and clear dialogue about the continuation of the partnership, clear expectations of when the partnership may end. |
| Transparency | Budgets and economic impact are publicly available as appropriate and shared among partners. |
| Partnership not program | Timelines and commitment extend beyond a single episode. |

Appendix 3: Information and Consent Forms

**Appendix 3.1: Information and Consent Form for Survey**

**IRB approval number:** UGHE-IRB/2024/293

**Project Title:** Assessing a Partnership-based Model Approach of Surgical Education at the University of Global Health Equity

| **Principal investigators** | Dr. Naol Belema Gemechu  [naol.belema@student.ughe.org](mailto:naol.belema@student.ughe.org)  Gatwiri Murithi  [gatwiri.murithi@student.ughe.org](mailto:Gatwiri.murithi@student.ughe.org) |
| --- | --- |
| Supervisor | Dr. Robert Riviello  [rriviello@ughe.org](mailto:rriviello@ughe.org)  Dr. Derbew Fikadu Berhe  [dfikadu@ughe.org](mailto:dfikadu@ughe.org)  Dr. Betel Amdeslassie Fenta  [afenta@ughe.org](mailto:afenta@ughe.org) |
| Preceptor: | Dr. Geoffery A. Anderson |

Version Number: Version 1  Version Date: March 26, 2024

We invite you to participate in a research study by Dr. Naol Gemechu and Gatwiri Murithi. You are being asked to participate in this study because you have been involved in surgical education programs conducted at UGHE. Participating in this research is entirely your choice. Your future opportunities with UGHE or its partners will remain the same if you decide not to participate.

The information below tells you about what is involved in the research, what you will be asked to do, and any benefit, risk, inconvenience, or discomfort you might experience. You can discuss any questions about this study with any member of the study team mentioned above. Please reach out via email and ask as many questions as you like.

**What is the study about?**

The study seeks to assess the partnership-based models used in the delivery of surgical education programs at UGHE. The study aims to understand the utility of this approach in enhancing knowledge transfer and learner performance in surgical education. It also seeks to evaluate the approach UGHE, and its partners used to ensure that the partnerships maintain mutual benefits for all parties involved while maintaining best practices that nurture equity. The findings will inform process improvement and document best practices for partnership development.

The specific objectives of this study as they relate to your involvement are to:

1. Describe what a successful partnership means for UGHE and its partners in surgical education.
2. Document the partnerships UGHE has engaged in surgical education programs within the last three years regarding geographic distribution, nature of organizations, duration of engagement, and areas of involvement.
3. Evaluate the perspectives of students, faculty, and partners on partnership-based surgical education programs implemented at UGHE in the last three years.

This study is important as UGHE extensively uses partnerships to build its internal capacity to deliver high-quality medical education programs and the expertise to deliver comprehensive, equitable health services.

**Who is doing the research?**

The study is being conducted by Dr. Naol Gemechu and Gatwiri Murithi (UGHE), supervised by Dr. Robert Riviello, Dr. Derbew Fikadu Berhe, and Dr. Betel Amdeslassie Fenta. Dr. Geoffrey A. Anderson will serve as the preceptor for the students. Dr. Naol and Gatwiri will use the results of this research study to form part of their capstone report to obtain a Master's in Global Health Delivery (Global Surgery).

There will be no costs to you, and you will not be paid for participating in this study.

**Why am I being asked to take part, and what will I have to do?**

You have been asked to take part because you are either currently or have been involved in the surgical education programs offered at UGHE. If you agree to participate, you can provide feedback through this online Survey. You have at least two weeks within which you can complete it at a convenient time.

**Are there any benefits to being in the research study?**

There may be no direct benefit to you from participating in this research. Your feedback will help strengthen the quality of UGHE’s partnerships and inform potential opportunities for growth and development. It will also inform best practices in equitable partnership models.

**Are there any risks, side effects, discomforts, or inconveniences from being in the research study?**

The main foreseeable risk is the time required to provide feedback through the Survey or interviews. The online survey will take only thirty minutes, and the one-on-one interviews will take only one hour.

**Who will have access to my information?**

Your demographic details and responses will be collected and stored electronically as part of the data collection. Your responses will be linked to a participant identification number, which will be de-identified prior to sharing with other researchers and analysis. Your information will be treated as confidential and used only for research purposes. The following people will have access to the de-identified information we collect in this research: the research team and, if there is an audit or investigation, staff from the UGHE Institutional Review Board.

Electronic data will be password-protected, and hard copy data will be in locked storage. The information collected in this study will be kept under secure conditions at UGHE for ten years after the research is published, and then it will be destroyed. The results of this research may be presented at conferences or published in professional journals.

**Will you tell me the results of the research?**

A summary of the findings will be shared once the data analysis and report writing are completed. The results will not be individual but based on all the information we collect and review as part of the research. Any publication or knowledge product produced from this study will also be shared with the participants.

**Do I have to take part in the research study?**

Taking part in a research study is entirely voluntary. It is your choice whether to take part or not. If you decide to take part and then change your mind, you can freely withdraw from the study. Your decision to participate or not in the study will not affect your relationship with UGHE.

You are free to withdraw from the study at any point.

**What happens next, and who can I contact about the research?**

To participate in this study, please use the shared survey link to provide your feedback. Clicking on the link and completing the Survey implies your consent to participate.

Please take your time and ask any questions you have before you decide what to do. You can reply to the principal investigators (Dr. Naol and Gatwiri) via email if you have any more questions. Likewise, you are welcome to contact the supervisor (Dr. Robert Riviello) or the University of Global Health Equity Institutional Review Board ([irb@ughe.org](mailto:irb@ughe.org), telephone: 0788316894 or Office of Human Research Administration (OHRA) at Kigali Heights Building, 5th floor, Kacyiru, Kigali, P.O. Box 6955, Rwanda.) for any concerns related to the conduct of this research.

Thank you for considering this study.

**Appendix 3.2: Information and consent form for Survey for interviews**

**IRB approval number:** UGHE-IRB/2024/293

**Project Title:** Assessing a Partnership-based Model Approach of Surgical Education at the University of Global Health Equity

| **Principal investigators** | Dr. Naol Belema Gemechu  [naol.belema@student.ughe.org](mailto:naol.belema@student.ughe.org)  Gatwiri Murithi  [gatwiri.murithi@student.ughe.org](mailto:Gatwiri.murithi@student.ughe.org) |
| --- | --- |
| Supervisors | Dr. Robert Riviello  [rriviello@ughe.org](mailto:rriviello@ughe.org)  Dr. Derbew Fikadu Berhe  [dfikadu@ughe.org](mailto:dfikadu@ughe.org)  Dr. Betel Amdeslassie Fenta  [afenta@ughe.org](mailto:afenta@ughe.org) |
| Preceptor: | Dr. Geoffery A. Anderson |

Version Number: Version 1  Version Date: March 26, 2024

We invite you to take part in a research study being conducted by Dr. Naol Gemechu and Gatwiri Murithi. You are being asked to be a part of this study because you have been involved in surgical education programs conducted at UGHE. Participating in this research is entirely your choice. Your future opportunities with UGHE or its partners will remain the same if you decide not to participate.

The information below tells you about what is involved in the research, what you will be asked to do, and about any benefit, risk, inconvenience, or discomfort that you might experience. You can discuss any questions you have about this study with any member of the study team mentioned above. Please reach out via email and ask as many questions as you like.

**What is the study about?**

The study seeks to assess the partnership-based models used in the delivery of surgical education programs at UGHE. The study aims to understand the utility of this approach in enhancing knowledge transfer and learner performance in surgical education. It also seeks to evaluate the approach used by UGHE and its partners to ensure that the partnerships maintain mutual benefits for all parties involved while maintaining best practices that nurture equity. The findings will inform process improvement and document best practices for partnership development.

The specific objectives of this study as they relate to your involvement are to:

1. Describe what a successful partnership means for UGHE and its partners in surgical education.
2. Document the partnerships UGHE has engaged in surgical education programs within the last three years regarding geographic distribution, nature of organizations, duration of engagement, and areas of involvement.
3. Evaluate the perspectives of students, faculty, and partners on partnership-based surgical education programs implemented at UGHE in the last three years.

This study is important as UGHE extensively uses partnerships to build its internal capacity to deliver high-quality medical education programs and the expertise to deliver comprehensive, equitable health services.

**Who is doing the research?**

The study is being conducted by Dr. Naol Gemechu and Gatwiri Murithi (UGHE), supervised by Dr. Robert Riviello, Dr. Derbew Fikadu Berhe, and Dr. Betel Amdeslassie Fenta. Dr. Geoffrey A. Anderson will serve as the preceptor for the students. Dr. Naol and Gatwiri will use the results of this research study to form part of their capstone report to obtain a Master's in Global Health Delivery (Global Surgery).

There will be no costs to you, and you will not be paid for participating in this study.

**Why am I being asked to take part, and what will I have to do?**

You have been asked to participate because you are either currently or have been involved in the surgical education programs offered at UGHE. If you agree to participate, you can provide feedback through this online Survey. You have at least two weeks within which you can complete it at a convenient time.

**Are there any benefits to being in the research study?**

There may be no direct benefit to you from participating in this research. Your feedback will help strengthen the quality of UGHE’s partnerships and inform potential opportunities for growth and development. It will also inform best practices in equitable partnership models.

**Are there any risks, side effects, discomforts, or inconveniences from being in the research study?**

The main foreseeable risk is the time required to provide feedback through the Survey or interviews. The online survey will take only thirty minutes, and the one-on-one interviews will take only one hour.

**Who will have access to my information?**

Your demographic details and responses will be collected and stored electronically as part of the data collection. Your responses will be linked to a participant identification number, which will be de-identified before sharing with other researchers and analysis. Your information will be treated as confidential and used only for research purposes. The following people will have access to the de-identified information we collect in this research: the research team and, if there is an audit or investigation, staff from the UGHE Institutional Review Board.

Electronic data will be password-protected, and hard copy data will be in locked storage. The information collected in this study will be kept under secure conditions at UGHE for ten years after the research is published, and then it will be destroyed. The results of this research may be presented at conferences or published in professional journals.

**Will you tell me the results of the research?**

A summary of the findings will be shared once the data analysis and report writing are completed. The results will not be individual but based on all the information we collect and review as part of the research. Any publication or knowledge product produced from this study will also be shared with the participants.

**Do I have to take part in the research study?**

Taking part in a research study is entirely voluntary. It is your choice whether to take part or not. If you decide to participate and then change your mind, you can freely withdraw from the study. Your decision to participate or not in the study will not affect your relationship with UGHE.

You are free to withdraw from the study at any point.

**What happens next, and who can I contact about the research?**

To participate in this study, please respond to this email to communicate your willingness to participate. The principal investigators will follow up to book an interview that can be done either in person or online.

Please take your time and ask any questions before deciding what to do. You can reply to the principal investigators (Dr. Naol and Gatwiri) via email if you have any more questions. Likewise, you are welcome to contact the supervisor (Dr. Robert Riviello) or the University of Global Health Equity Institutional Review Board ([irb@ughe.org](mailto:irb@ughe.org), telephone: 0788316894 or Office of Human Research Administration (OHRA) at Kigali Heights Building, 5th floor, Kacyiru, Kigali, P.O. Box 6955, Rwanda.) for any concerns related to the conduct of this research.

Thank you for considering this study.

Statement of consent

I have read the information in this consent form, including risks and benefits. All my questions about the research have been answered to my satisfaction. I understand that I can withdraw at any time without penalty or loss of benefits to which I am otherwise entitled.

I consent to participate in the study.

Your signature below indicates your permission to take part in this research.

|  |  |  |
| --- | --- | --- |
| Name of participant | | |
|  |  |  |
| Signature of participant |  | Date |
|  |  |  |
| Signature of the person obtaining consent |  | Date |

Appendix 4: Interview Guide

**Appendix 4.1: Key Informant Interview Guide-UGHE**

Participant ID:

| **Thematic area** | **Main question** | **Probing questions** |
| --- | --- | --- |
| Demographics | Briefly describe your role in surgical education at UGHE | Age, Gender, Profession, Service year, and current role at UGHE |
| Motivations | How do you define a partnership-based model?   Why did you adopt it in the delivery of surgical education? | - What gaps were you trying to mitigate? - How did partners support/fill the gap? - What factors made you consider this approach? - What do you look for in a good partner? |
| Processes | Can you describe how the partnerships were formed? | - Who initiated the partnership? - How did the initial communication happen? - How do you develop common goals? - How do you address power imbalances? - Please give an example where you would say no to a potential partner |
| Benefits | Please describe what you perceive to be a good partnership. Do you have an example of one? | - What made them good partners? - How did you maintain a good working relationship? - How were conflicts resolved in your partnership? - Was there any institutional change that came because of these partnerships? - How did you decide on sharing roles and responsibilities? |
| Challenges | Please describe what you perceive to be a bad partnership. Do you have an example of one? | - Have internal processes been developed to mitigate such challenges? - Is there a mechanism for partners to report and discuss challenges? |
| Effectiveness | What metrics would you use to define an effective partnership? | - What metrics/indicators do you use at the individual (students and faculty), institutional, and system levels? - Are there any strategies you use to enhance effectiveness? - How do you sustain good results? - What indicators do you use to define failure? |
|  | How do programs delivered independently by UGHE differ from those delivered in partnerships? | - What are the pros and cons of each? |
| Equity | What do you do to ensure equity in the partnerships you are engaged in? | - What factors do you feel promote/limit equity? - How are power dynamics managed in partnership? |

**Appendix 4.3: In-Depth Interview Guide-Students**

**Participant ID:**

| **Thematic area** | **Main question** | **Probing questions** |
| --- | --- | --- |
| Demographics | Briefly describe yourself and your experience in surgical education programs at UGHE. | - When did you join UGHE? - Please describe any training you have received in surgery, anesthesia, and obstetrics/gynecology |
| Perception | Can you describe any training surgical training sessions facilitated by visiting faculty? | - What was your favorite experience, and why did you like it? - What was your worst experience, and why did you dislike it? - How could it be made better? |
| Effectiveness | How did the visiting faculty affect your learning? | - Did you have enough time to learn from the visiting faculty? - Was the material taught easy or challenging to learn? - Do you feel confident about applying what you learned from the visiting faculty? |
|  | Please describe your interaction with the visiting faculty. | - Did you have any challenges interacting with them? If yes, please explain |

**Appendix 4.4: In-Depth Interview Guide-UGHE Faculty**

**Participant ID:**

| **Thematic area** | **Main question** | **Probing questions** |
| --- | --- | --- |
| Demographics | Briefly describe yourself and your experience in surgical education programs at UGHE. | - Please describe any training you have given in surgery, anesthesia, and obstetrics/gynecology. When did you start engaging with UGHE? - Are you involved in both didactics and practical training? |
| Perception | Can you describe any surgical training sessions you have been involved with visiting faculty? | - What was your favorite experience, and why did you like it? - What was your worst experience, and why did you dislike it? - How could it be made better? - How were the roles divided between the facilitators? - Did you feel well-engaged during this session? |
| Effectiveness | From your experience, how have the partners involved in surgical education affected learning outcomes at UGHE? | - Did you have enough time with each partner? - Was the material taught relevant to the African context? - Has the interaction with them improved how you teach? |
|  | From your experiences, how have partners affected your capacity as a trainer at UGHE? |  |
|  | Please describe your interaction with UGHE's partners. | - Did you have any challenges interacting with them? If yes, please explain. - Please share an example of a positive interaction |
| Equity | In your view, has equity been achieved in the partnerships UGHE has engaged in? | - How has responsibility been shared? - Has this distribution been fair? - Were you included in setting up joint learning sessions? - Was there a mechanism to report and resolve conflict? - How were potential power imbalances mitigated? |

**Appendix 4.5: In-Depth Interview Guide-UGHE partners**

**Participant ID:**

| **Thematic area** | **Main question** | **Probing questions** |
| --- | --- | --- |
| Demographics | Briefly describe yourself and your experience in surgical education programs at UGHE. | - Please describe any training you have given in surgery, anesthesia, and obstetrics/gynecology. When did you start engaging with UGHE? - Are you involved in both didactics and practical training? |
| Perception | Can you describe any surgical training session you have been involved in at UGHE? | - What was your favorite experience, and why did you like it? - What was your worst experience, and why did you dislike it? - How could it be made better? - Did you feel well-engaged during this session? |
| Effectiveness | From your experience, how has your involvement in surgical education affected learning outcomes at UGHE? | - Did you have enough time to cover all your material? - How did the students and faculty respond to your training? |
|  | Please describe your interaction with the UGHE community (students and faculty). | - Did you have any challenges interacting with them? If yes, please explain. - Please share an example of a positive interaction |
| Equity | In your view, has equity been achieved in the partnerships UGHE has engaged in? | - How has responsibility been shared? - How were decisions made in the partnerships? - Was there a mechanism to report and resolve conflict? - How were potential power imbalances mitigated? |

Appendix 5: Study Survey Tool

**Appendix 5.1: Student questionnaire**

| **Student Level** | |  | 4^th^ -year/ MBBS ‘26 | |  | | | 5^th^ year/MBBS ‘25 | |  | | | MGHD (Global surgery) | | | | | |  |
| --- | --- | --- | --- | --- | --- | --- | --- | --- | --- | --- | --- | --- | --- | --- | --- | --- | --- | --- | --- |
| **Which of the following learning activities have you engaged in in the last 12 months that were facilitated by visiting faculty?** (Mark all that apply) | | | | | | | | | | | | | | | | | | | |
|  | Skills Workshops | | |  | | | Simulation sessions | | | |  | | | Webinars | |  | | | |
|  | Clinical training | | |  | | | Classroom Lectures | | | |  | | |  | |  | | | |
| **Please indicate your level of agreement with the following statements. (Unless otherwise stated, all statements refer to activities facilitated by visiting faculty)** | | | | | | | | | | | | | | | | | | |  |
|  | | | | | | | **Strongly disagree** | | | **Disagree** | | | **Neither agree nor disagree** | | | **Agree** | | **Strongly Agree** |  |
| The didactic surgical education sessions aided my understanding and engagement with the course material. | | | | | | |  | | |  | | |  | | |  | |  |  |
| The clinical/simulation surgical education sessions aided my understanding and engagement with the course material. | | | | | | |  | | |  | | |  | | |  | |  |  |
| I had a good understanding of the course material from the sessions facilitated by visiting faculty. | | | | | | |  | | |  | | |  | | |  | |  |  |
| I had adequate time to learn through the sessions facilitated by visiting faculty. | | | | | | |  | | |  | | |  | | |  | |  |  |
| The visiting faculty improved my overall training experience. | | | | | | |  | | |  | | |  | | |  | |  |  |
| The visiting faculty worsened my overall training experience. | | | | | | |  | | |  | | |  | | |  | |  |  |
| I am comfortable with the treatment I have received from visiting faculty | | | | | | |  | | |  | | |  | | |  | |  |  |
| I am comfortable with the treatment I have received from visiting faculty | | | | | | |  | | |  | | |  | | |  | |  |  |
| I am comfortable with the treatment I have received at the teaching hospitals (clinical sites) | | | | | | |  | | |  | | |  | | |  | |  |  |
| I feel comfortable communicating with visiting faculty | | | | | | |  | | |  | | |  | | |  | |  |  |
| I feel comfortable communicating with staff at the teaching hospitals | | | | | | |  | | |  | | |  | | |  | |  |  |
| I feel comfortable raising concerns with visiting faculty | | | | | | |  | | |  | | |  | | |  | |  |  |
| I feel comfortable raising concerns at the teaching hospitals. | | | | | | |  | | |  | | |  | | |  | |  |  |
| I feel comfortable asking questions to visiting faculty | | | | | | |  | | |  | | |  | | |  | |  |  |
| I feel comfortable asking questions to staff at the teaching hospitals | | | | | | |  | | |  | | |  | | |  | |  |  |
| I feel heard by visiting faculty | | | | | | |  | | |  | | |  | | |  | |  |  |
| I feel heard by the staff at the teaching hospitals. | | | | | | |  | | |  | | |  | | |  | |  |  |
